# On Lead-Poisoning

**Published:** 1870-01

**Authors:** 


### On Lead-Poisoning.

Dr. Edward Hitzig of Berlin (*The Quart. Jour. of Psychological Medicine*), after a comprehensive survey of lead-poisoning, lays down the subjoined conclusions:

1. Lead, after its absorption into the circulation, may remain in the organization for a long time without exhibiting its characteristic symptoms of poisoning.

2. When these symptoms appear, they are to be regarded as a result of an expurgation of the lead from the circulation into the organs; and indeed those symptoms are (excepting the encephalic) made manifest mostly in the muscles, through direct lesion of the capillary system.

3. A fixed lead concentration in the blood is not the only necessary precursor of symptoms of lead-poisoning, but rather indicates certain affections of the coats of blood-vessels, which interfere materially with the normal process of exudation.

4. The afore-mentioned affection of the blood-vessels exists, during the first stage, in a more powerful contraction of the muscles and a reduction of their calibre; consequently, the intravascular pressure, and with it the transudation, is increased, so that a reduction in the volume of blood ensues through a loss of a portion cf its watery substance. The opposition noticed in the circulation from the same cause may be the preparatory symptom of local-organ poisoning.

5. During the second stage we noticed relaxation, and a varicose degeneration of the venous coats, rendering the valve insufficient. Even these changes assist through the possibility of the lead compounds passing into organs.

6. The peculiar predisposition of certain muscles to lead paralysis manifests itself through the various conditions of the veins of those muscles.

7. The manufacture of curled hair, and undoubtedly the use of poor materials, predisposes to lead-poisoning.

8. Mobility in cases of lead-poisoning, is lost before the electric contractility.—*Med. Review.*

